# Lasing in Two-Dimensional Tin Perovskites

**DOI:** 10.1021/acsnano.2c07705

**Published:** 2022-11-24

**Authors:** Ada Lilí Alvarado-Leaños, Daniele Cortecchia, Christian Niclaas Saggau, Samuele Martani, Giulia Folpini, Elena Feltri, Munirah D. Albaqami, Libo Ma, Annamaria Petrozza

**Affiliations:** † Istituto Italiano de Tecnologia, Centre for Nano Science and Technology (CNST@PoliMi), Milan20133, Italy; ‡ Physics Department, Politecnico di Milano, Piazza Leonardo da Vinci 32, Milan20133, Italy; § Institute for Integrative Nanosciences, Leibniz IFW Dresden, Dresden01069, Germany; ∥ Chemistry Department, College of Science, King Saud University, Riyadh11451, Saudi Arabia

**Keywords:** two-dimensional perovskites, tin perovskites, lasing, ASE, DFB laser

## Abstract

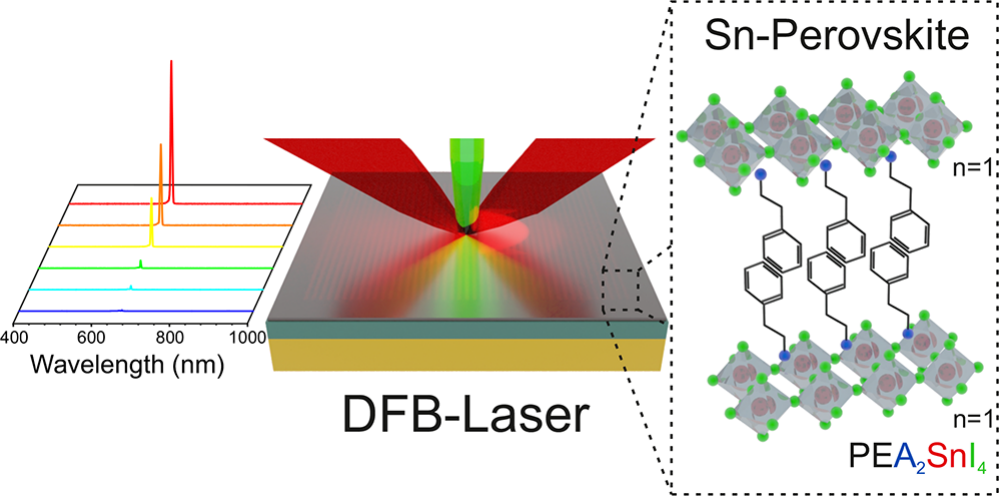

Two-dimensional (2D) perovskites have been proposed as materials capable of improving the stability and surpassing the radiative recombination efficiency of three-dimensional perovskites. However, their luminescent properties have often fallen short of what has been expected. In fact, despite attracting considerable attention for photonic applications during the last two decades, lasing in 2D perovskites remains unclear and under debate. Here, we were able to improve the optical gain properties of 2D perovskite and achieve optically pumped lasing. We show that the choice of the spacer cation affects the defectivity and photostability of the perovskite, which in turn influences its optical gain. Based on our synthetic strategy, we obtain PEA_2_SnI_4_ films with high crystallinity and favorable optical properties, resulting in amplified spontaneous emission (ASE) with a low threshold (30 μJ/cm^2^), a high optical gain above 4000 cm^–1^ at 77 K, and ASE operation up to room temperature.

Metal halide perovskites are interesting and promising materials for photonic applications given their synthetic flexibility and good optoelectronic properties.^1,2^ Since the first demonstration of amplified spontaneous emission (ASE) and lasing from methylammonium lead halides,^3,4^ the coherent emission properties of three-dimensional (3D) perovskites have been extensively investigated. In addition, it has been demonstrated that a variety of resonators can be employed to fabricate perovskite lasers.^5−9^ However, the current research has evidenced the need to overcome the detrimental nonradiative losses typical of 3D perovskites, along with increasing their radiative recombination efficiency and stability. Moreover, it is still of fundamental importance to find efficient and stable nontoxic alternatives to lead-based compositions. All considered, two-dimensional (2D) perovskites could be alternative materials to enhance the luminescence efficiency, given their high exciton binding energy, which stems from their stable excitons with fast radiative decay.^10−16^ Additionally, the layered architecture of 2D perovskites have enabled an improved stability^17^ as well as a rich chemical diversity, which can allow to circumvent lead compositions.^18^ Although back in 1998 lasing was reported in PEA_2_PbI_4_ (PEA = phenetylammonium) at 16 K,^19^ those results left a series of open questions due to the difficulties in reproducibility. Consequently, the possibility to sustain coherent emission in 2D perovskites has remained unclear and under debate, especially for the lowest-dimensional member (*n* = 1) of the Ruddlesden–Popper series (RNH_3_)_2_(A)_*n*−1_[M_*n*_X_3*n*+1_].^20−22^ Several studies have shown an increase in optical losses as the dimensionality is decreased and have estimated that the ASE threshold in *n* = 1 2D perovskites exceeds their damage threshold.^20−22^ In contrast, it was found that lasing in DA_2_PbI_4_ (DA = dodecylammonium) could take place below 125 K,^23^ while lasing and random lasing have been observed in BA_2_PbI_4_ single crystals and exfoliated PEA_2_PbI_4_ flakes.^24,25^ Even though lasing has been claimed in these systems, to the best of our knowledge amplified spontaneous emission in 2D perovskites has never been published. This is a critical issue, since studying ASE can help to understand how the optical gain characteristics of the material, are affected by its structure and composition. For example, the presence of ASE can give information about key parameters, such as the light amplification per unit length of the semiconductor, which in turn assesses their suitability for lasing as compared to other gain media.^26^ The study of lasing in low-dimensional perovskites has focused on Pb-based materials, while 2D Sn perovskites research has mostly centered around charge transport. For example, 2D Sn perovskites have been used as semiconducting channel materials for field-effect transistors enabling both p- and n-type transport.^27−29^ In addition, 2D Sn perovskites have been employed as active materials in light-emitting diodes and have achieved a record external quantum efficiency of 5%, thus outperforming lead-based 2D perovskites.^30,31^ Recently, we have studied the transient absorption spectral features in PEA_2_SnI_4_, which can be attributed to stimulated emission, suggesting its potential as a gain medium.^32^ 2D tin perovskites, aside from representing a greener alternative to their Pb counterparts, can also be interesting for its integration in planar device architectures, as they possess a combination of good in-plane charge transport, notable light-emitting properties and suppressed ionic migration. This planar architecture is relevant for electrically pumped lasing given its minimized optical losses.^29,33,34^ However, to take advantage of the planar architecture for lasing, it is crucial to tune the optical gain properties of 2D perovskites, which could be achieved through synthetic material design.

In this work, we investigate the ASE of three different 2D tin perovskites, BA_2_SnI_4_, PEA_2_SnI_4_, and NMA_2_SnI_4_, where the bulkiness of the spacer cation is progressively increased from butylammonium (BA) to phenethylammonium (PEA) and 1-naphthylmethylammonium (NMA). The change of molecular geometry and nature of the intermolecular forces holding the crystal (van der Waals forces in BA, and π–π interactions in PEA and NMA) affect the structural rigidity, crystallinity, and defectivity, with considerable consequences on their photophysical properties and ability to sustain ASE. The synergy of these factors results in the highest optical quality and photostability for PEA_2_SnI_4_, where we probed ASE up to room temperature. At 77 K, this material shows a low-threshold ASE, down to 30 μJ/cm^2^, and high optical gain beyond 4000 cm^–1^. Taking advantage of these ASE characteristics, we integrate PEA_2_SnI_4_ in a distributed feedback (DFB) resonator, designed *ad hoc* to match the gain spectrum of the material. With the final device we were able to demonstrate that 2D tin perovskites can act as promising gain media for lasing.

## 2 Results and Discussion

**Figure 1:**
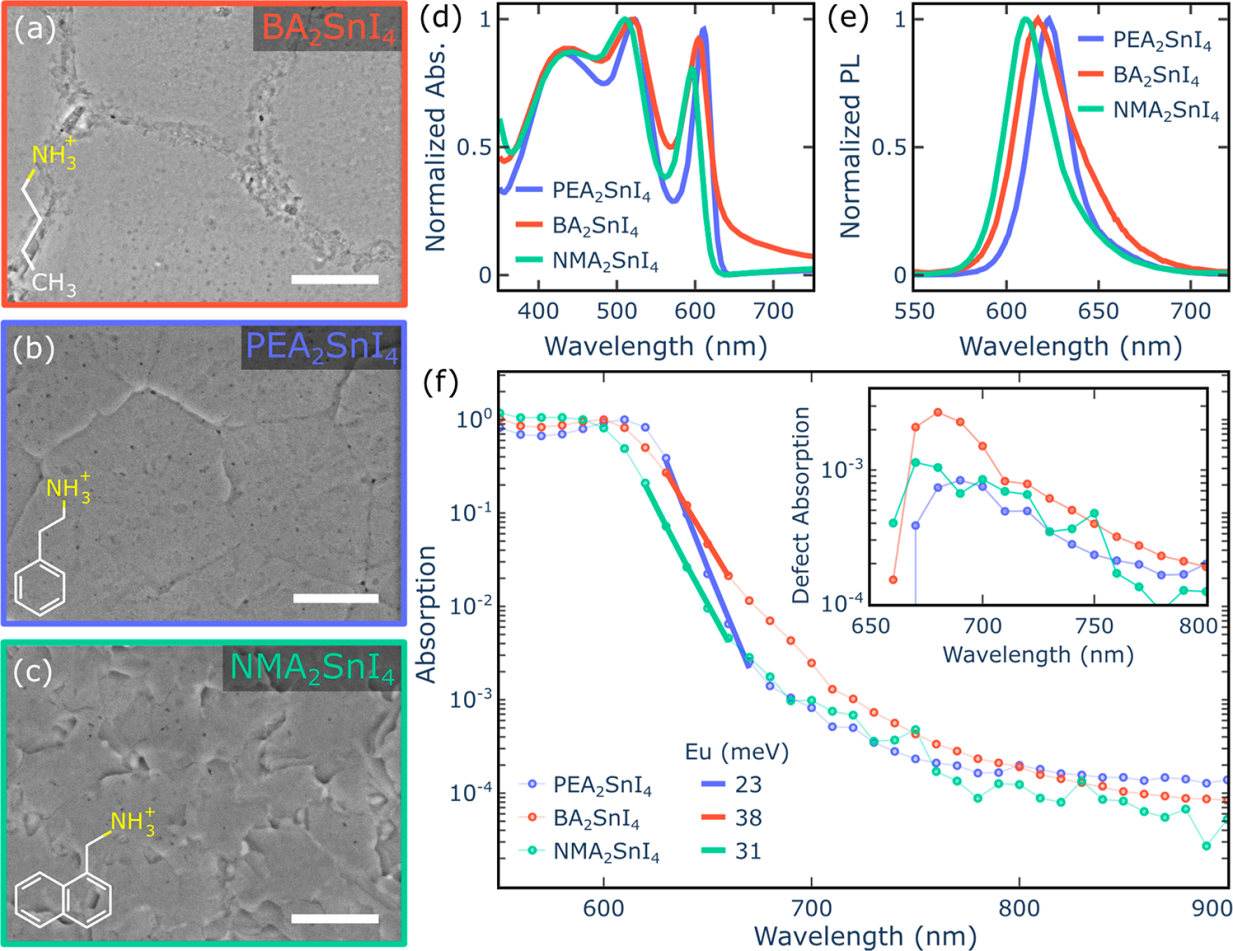
Scanning electron microscope (SEM) images show the crystallization morphologies of three different perovskites: BA_2_SnI_4_ (a), PEA_2_SnI_4_ (b), and NMA_2_SnI_4_ (c) (scale bar = 2 μm). The absorption (d) and photoluminescence spectra (e) for these three perovskites are also presented. (f) Photothermal deflection spectra comparing the Urbach tails of these three materials to the corresponding Urbach Energy (*E*_U_). The inset highlights the defect absorption by subtracting the fitted Urbach tail from the pristine spectra. All results correspond to room temperature measurements.

The formation of solution-processed perovskite thin films was confirmed by X-ray diffraction (XRD), indicating an increase of the interplanar distance of the perovskite as the cation size increased (Figure S1). Through temperature-dependent XRD we determined the thermal expansion coefficient α (Figure S1),^35,36^ which is closely linked to the structural rigidity.^37,38^ For BA_2_SnI_4_, where the aliphatic chain of BA is highly mobile,^14^ we obtained α = 154 × 10^–6^ K^–1^, suggesting a high structural flexibility. In fact, its lattice undergoes a contraction of about 7% down to 78 K, mediated by a first-order phase transition, which takes place at 220 K and results in a more tightly packed low-temperature phase with α = 102 × 10^–6^ K^–1^.^39^ In contrast to the effect induced by the aliphatic chain of BA, the aromatic cores of PEA and NMA provide a higher structural rigidity to PEA_2_SnI_4_ (α = 94 × 10^–6^ K^–1^) and NMA_2_SnI_4_ (α = 92 × 10^–6^ K^–1^), thus stabilizing their crystal structure across the temperature range 298–80 K, with a smaller 2% contraction of their interplanar distance. Our retrieved values are higher compared to those reported for 3D perovskites (α = 28 × 10^–6^ K^–1^ for CsPbBr_3_),^36^ indicating the importance of the nature of the spacer cation in determining the structural rigidity of the perovskite lattice.^14^ In addition, the role of the chemical composition on the crystallinity and microstructure of the film was observed. For example, BA_2_SnI_4_ showed a considerably weaker diffraction intensity compared to the other two perovskites (Figure S1), suggesting that the aromatic cores influence the formation of ordered and less defective crystal domains. Indeed, BA_2_SnI_4_ forms large domains broken by discontinuous and defective grain boundaries, while both PEA_2_SnI_4_ and NMA_2_SnI_4_ form compact films of large crystal grains with sizes exceeding 2 μm, showing well-defined polygonal morphologies and jagged textures, respectively (Figure 1a–c). The absorption spectra of the three perovskites possess similar features (Figure 1d), consisting of a sharp excitonic peak followed by the absorption continuum for wavelengths below 550 nm. The excitonic peak blue shifts from 611 nm to 596 nm in the following order: PEA_2_SnI_4_ > BA_2_SnI_4_ > NMA_2_SnI_4_. Previous works have shown that the variation of the bandgap and the absorption onset are closely linked to the changes in the structural properties of the perovskite, given that distorted geometries can decrease the width of the valence and conduction bands, thus increasing the bandgap.^39−41^ Several parameters can affect the energetic landscape, including the Sn–I–Sn bond angles, in-plane and out-of-plane octahedral tilt, Sn–I bond distance, and penetration depth of the organic cation. Since the crystal structure of NMA_2_SnI_4_ is unknown, it is not possible to identify which of these factors has the most significant influence on the widening of its bandgap. Nevertheless, the trend of the excitonic peak shift, observed in Figure 1d, indicates that NMA_2_SnI_4_ possesses an overall highly distorted coordination geometry in comparison to the other two perovskites, which agrees with the highest steric impact of its spacer cation. A similar pattern is present in the photoluminescence (PL), which blue shifts from 623 nm to 616 nm (Figure 1e). BA_2_SnI_4_ shows a broader PL bandwidth and more than 1 order of magnitude drop in intensity compared to PEA_2_SnI_4_ (Figure S2), suggesting the presence of a greater trap density. In particular, from the photothermal deflection spectroscopy (PDS) measurements presented in Figure 1f, the overall level of disorder can be estimated by fitting the Urbach Energy (*E*_U_) parameter. *E*_U_ was extracted from the sub-bandgap absorption below the main exciton line, which includes both the broadening of the exciton line due to disorder and the direct absorption of intergap states.^42^ The observed increase of *E*_U_ going from PEA_2_SnI_4_ (*E*_U_ = 23 meV) to NMA_2_SnI_4_ (*E*_U_ = 31 meV) and BA_2_SnI_4_ (*E*_U_ = 38 meV) confirms the notable optical quality of PEA_2_SnI_4_ in contrast to the highly defective BA_2_SnI_4_. In addition, the absorption tail from BA_2_SnI_4_ (Figure 1f) exhibits a bump centered at 680 nm, indicating the presence of a high concentration of shallow defect states at about 17 meV from the band edge. At low temperatures, around 220 K, BA_2_SnI_4_ shows an abrupt 40 nm blue shift of the excitonic emission and absorption (Figure S3), which agrees with the phase transition found by temperature-dependent XRD.^39^ Otherwise, PEA_2_SnI_4_ and NMA_2_SnI_4_ show a continuous monotonic red shift with a decrease in temperature (related to lattice contraction, Figure S1) of the PL, which progressively narrows and reveals a well-resolved excitonic fine structure (Figure S3).

**Figure 2:**
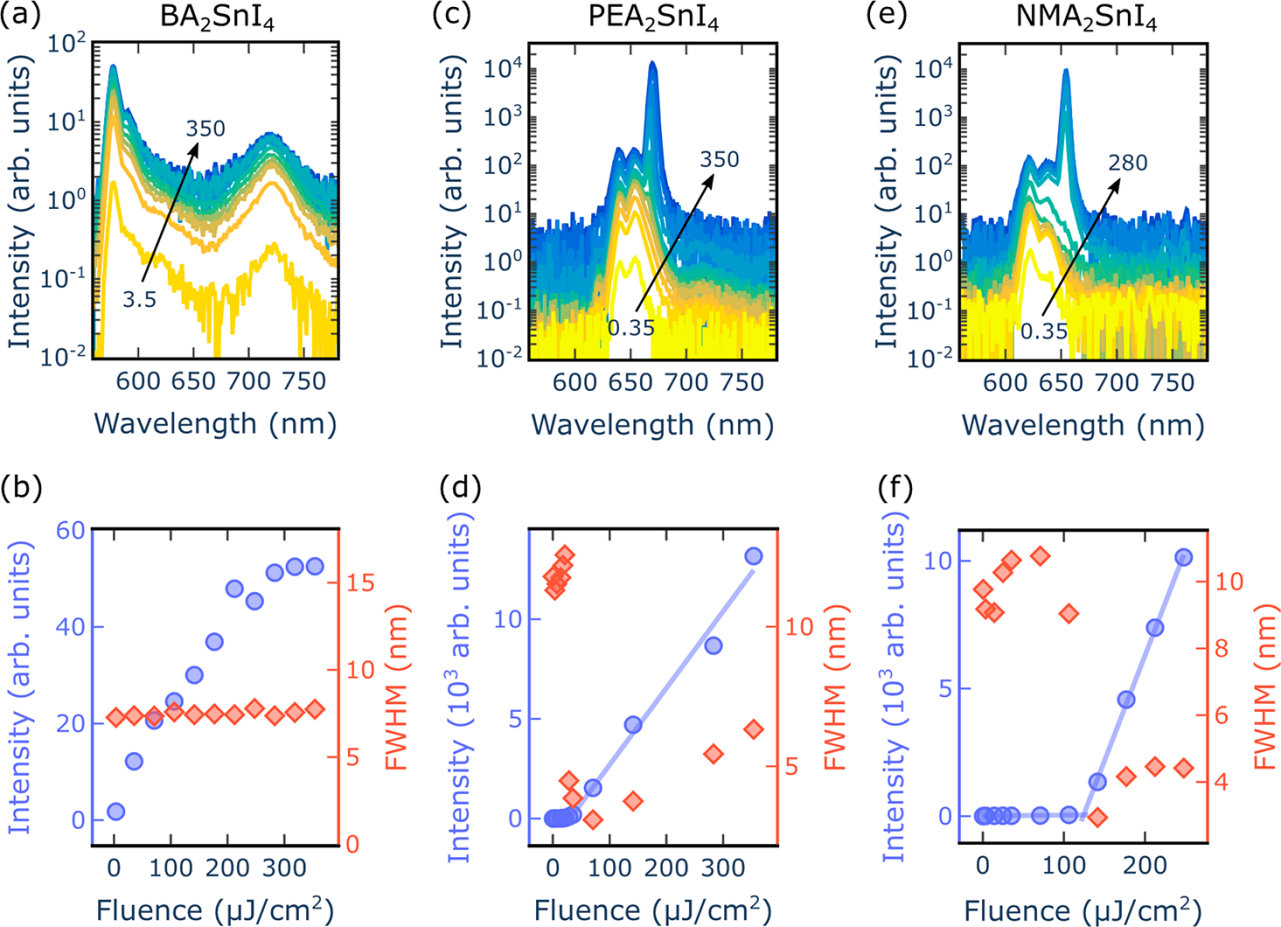
Fluence-dependent PL measurements at 77 K for BA_2_SnI_4_ (a, b), PEA_2_SnI_4_ (c, d) and NMA_2_SnI_4_ (e, f) are presented. Panels a, c, and e correspond to the fluence-dependent spectra, where the arrows indicate the fluence range in μJ/cm^2^. Panels b, d, and f show the peak intensity (blue) and full width at half-maximum (fwhm, red) evolution as a function of the excitation fluence (λ_exc_ = 532 nm, pulse duration 800 ps, repetition rate 1 kHz). In panel b, the intensity and fwhm were obtained considering the main excitonic peak of the BA_2_SnI_4_ spectra at around 577 nm.

With the objective of determining the presence of optical gain in PEA_2_SnI_4_, BA_2_SnI_4_, and NMA_2_SnI_4_, their ASE performance was studied. ASE takes place when spontaneously emitted photons propagate in an inverted gain medium and, in the process, stimulate the emission of additional photons. When carrying out fluence-dependent PL measurements on a sample that shows ASE, two responses as a function of the excitation pump intensity can be distinguished. For low pump intensities, only spontaneous emission can be observed, which is defined by a linear increase of the output intensity. Meanwhile, when high enough pump intensities are reached, ASE will dominate and manifest as a superlinear increase of the emitted intensity and a narrowing of the bandwidth. Figure 2 shows the fluence-dependent PL measurements of PEA_2_SnI_4_, BA_2_SnI_4_, and NMA_2_SnI_4_, performed at 77 K, under picosecond laser excitation.

The spectrum of BA_2_SnI_4_ (Figure 2a) presents a main excitonic emission, as well as a low-energy broad PL band from 680 nm to 750 nm, which matches the spectral range of the defect state observed in the absorption of Figure 1f. Due to the shallow nature of the traps, their emission is more easily observed at low temperature, where thermally activated detrapping is less likely to occur. Those defects compete with the main excitonic transitions, introducing losses that hamper light amplification. Overall, it was not possible to observe ASE from excitonic recombination in BA_2_SnI_4_ (Figure 2b), whereas the fluence-dependent measurements of PEA_2_SnI_4_ and NMA_2_SnI_4_ show a clear threshold behavior with a superlinear increase onset at 30 μJ/cm^2^ and 123 μJ/cm^2^, respectively (Figure 2c–f). Considerable spectral narrowing is observed as given by the appearance of an ASE peak with a fwhm of 3 nm, which develops on the red side of the spectra around 666 and 653 nm for PEA_2_SnI_4_ and NMA_2_SnI_4_, respectively. Despite having similar ASE performances, their ASE stability is notably different, as can be seen in Figure S4, where under an excitation fluence of 280 μJ/cm^2^ at 532 nm, the ASE of NMA_2_SnI_4_ completely quenches within the first 5 s of illumination, while the ASE of PEA_2_SnI_4_ remains unaltered even after 60 s. A similar trend is observed for the spontaneous emission, where the quenching is not reversible in the dark (Figures S5–S7). The significant change in the luminescence of NMA_2_SnI_4_ is accompanied by a more modest change in absorption, with a 10% bleach of the excitonic absorption after 90 s of light exposure (Figure S8). These results indicate that defects are quickly formed in the material, leading to its permanent photodegradation. Previous studies have shown that distortions of the I–M–I bond angles (M = Pb^2+^, Sn^2+^) play an important role in mediating the photodecomposition process of the perovskite.^43,44^ The more distorted coordination geometry of the SnI_6_ octahedra in NMA_2_SnI_4_ compared to PEA_2_SnI_4_ (as deduced from the data in Figure 1) can therefore be connected to its faster degradation. Moreover, the large molecular cross section of NMA implies that its flip motion induced by the resonant photoexcitation can be particularly disruptive for the local coordination geometry, thus inducing a higher rotational disorder of the SnI_6_ octahedral network giving more easily breakable Sn–I bonds and favoring the collapse of the perovskite framework.^43−45^ Therefore, even though NMA_2_SnI_4_ is initially characterized by low defectivity and has similar properties to PEA_2_SnI_4_, its pronounced photoinstability inhibits a stable ASE operation.

**Figure 3:**
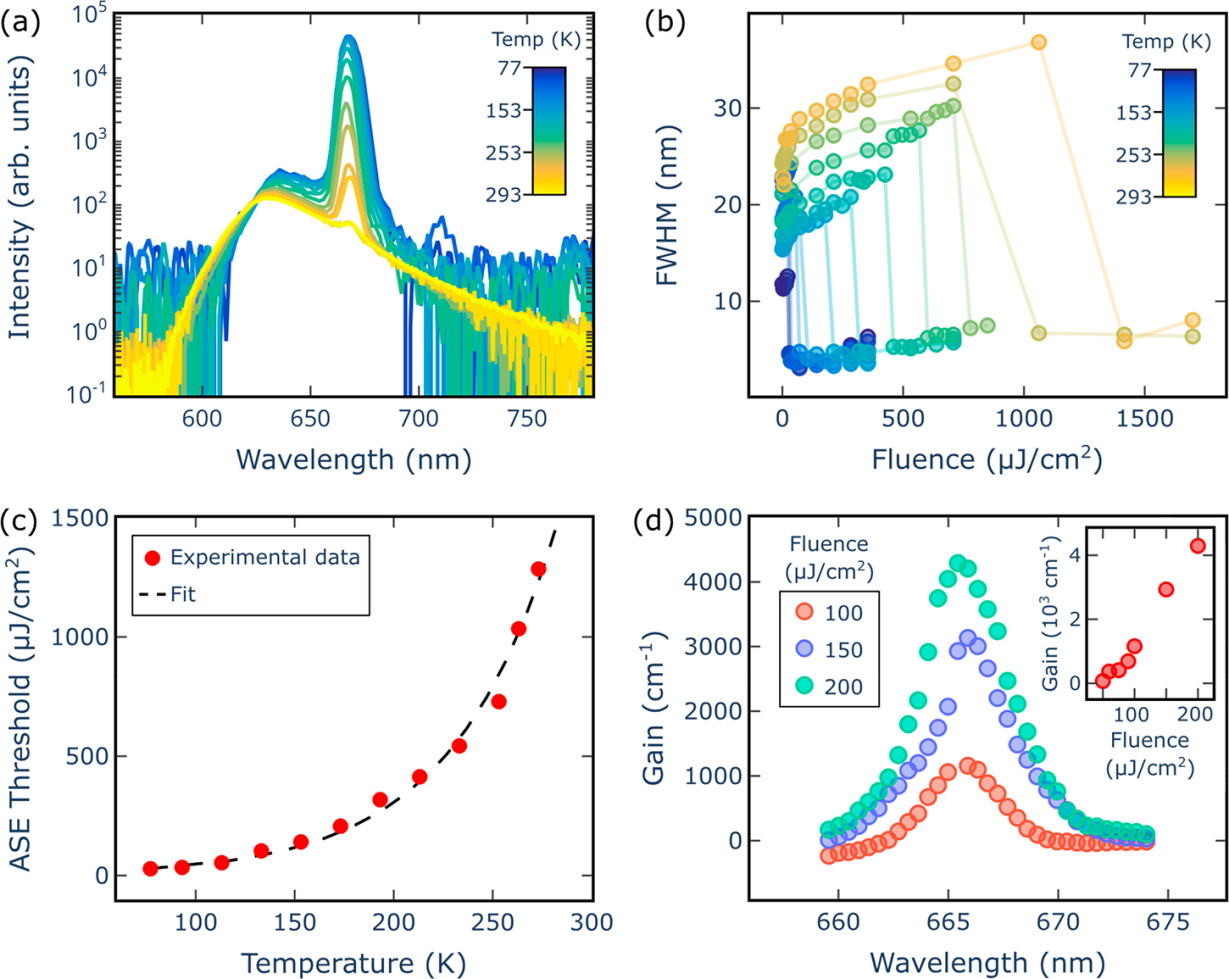
The optical gain properties of PEA_2_SnI_4_ are presented as follows: (a) temperature-dependent spectra above threshold, for an excitation fluence of 2 mJ/cm^2^ and (b) the corresponding change of the fwhm of the ASE peak; (c) the ASE threshold as a function of temperature, where the experimental data (red dots) are fitted (dashed black line) according to an exponential trend, from which a characteristic temperature *T*_0_ of 52 K can be extracted; and (d) the modal gain spectra for 3 different fluences and the fluence-dependent modal gain (inset), both at 77 K.

Considering the superior ASE performance of PEA_2_SnI_4_, we decided to focus on investigating its gain properties in order to assess the suitability of 2D tin perovskites for lasing. From temperature-dependent ASE measurements (Figure 3a and Figure S9), it was found that increasing the temperature resulted in a decrease of the ASE intensity and a broadening of the fwhm from 3 nm to 7 nm. (Figure S10). Although the ASE signal becomes much weaker approaching 293 K, it was still possible to probe its onset at room temperature, which is also confirmed by the reduction of the fwhm at 293 K (Figure 3b). Such a considerable thermal dependence is further evidenced by the clear decrease of the ASE slope intensity versus the excitation fluence present at high temperatures, while the ASE increases only slightly above 200 K (Figure S10 and Figure S11). In a previous work we showed that above 200 K, the large thermal phonon population becomes a dominant factor, resulting in a drop of the photoluminescence quantum yield of PEA_2_SnI_4_, which could similarly affect the ASE slope behavior.^46^ The temperature dependence of the ASE threshold can be described by the exponential function , where *F*_0_ is the threshold fluence approaching 0 K and *T*_0_ is known as the “characteristic temperature” (Figure 3c).^47−49,51−53^ Fitting the data plotted in Figure 3c gives *T*_0_ = 52 K, which is lower than the *T*_0_ typically measured for inorganic semiconductors such as CdSe and InGaAlAs, where *T*_0_ can exceed 100 K.^51−53^ This is consistent with the soft nature of the perovskite lattice, confirmed by temperature-dependent XRD, where thermal vibrations and high exciton–phonon coupling can easily introduce nonradiative recombination pathways, which diminish the gain buildup.

To further characterize the optical gain of PEA_2_SnI_4_ we employed the variable stripe length method (VSLM, see Methods for a more detailed description of the experimental technique). Here, a narrow stripe-shaped laser beam illuminates a section of the perovskite film, which acts as a waveguide and gives rise to a single pass amplification of photons. The length of the stripe is progressively increased, and the output intensity is collected at the edge of the sample. When plotting the output intensity as a function of the stripe length for a material that shows optical gain, stimulated emission will give rise to an exponential increase of the output intensity. Subsequently, at long enough stripe lengths, saturation of the stimulated emission takes place and manifests as a deviation from the initial exponential growth (Figure S12). By analyzing the VSL curve, the optical gain of PEA_2_SnI_4_ can be extracted.^26^ When increasing the pump fluence, the optical gain increases linearly, up to 4200 cm^–1^, with no signs of saturation for the measured fluence range (inset of Figure 3d). Moreover, from the VSL curves taken at different wavelengths, it was possible to retrieve the wavelength-dependent optical gain (Figure 3d). The resulting gain spectra are centered around 666 nm with fwhm = 3 nm, where no substantial shift of the peak intensity is observed when increasing the pump fluence.^26^ The obtained narrow bandwidth, even at high pump fluences, could help sustain a large density of population inversion concentrated in a narrow spectral region, thus allowing for a high optical gain.^49,50^ These notable optical gain values, comparable to MAPbI_3_,^26^ indicate that PEA_2_SnI_4_ can be an attractive lasing material.

**Figure 4:**
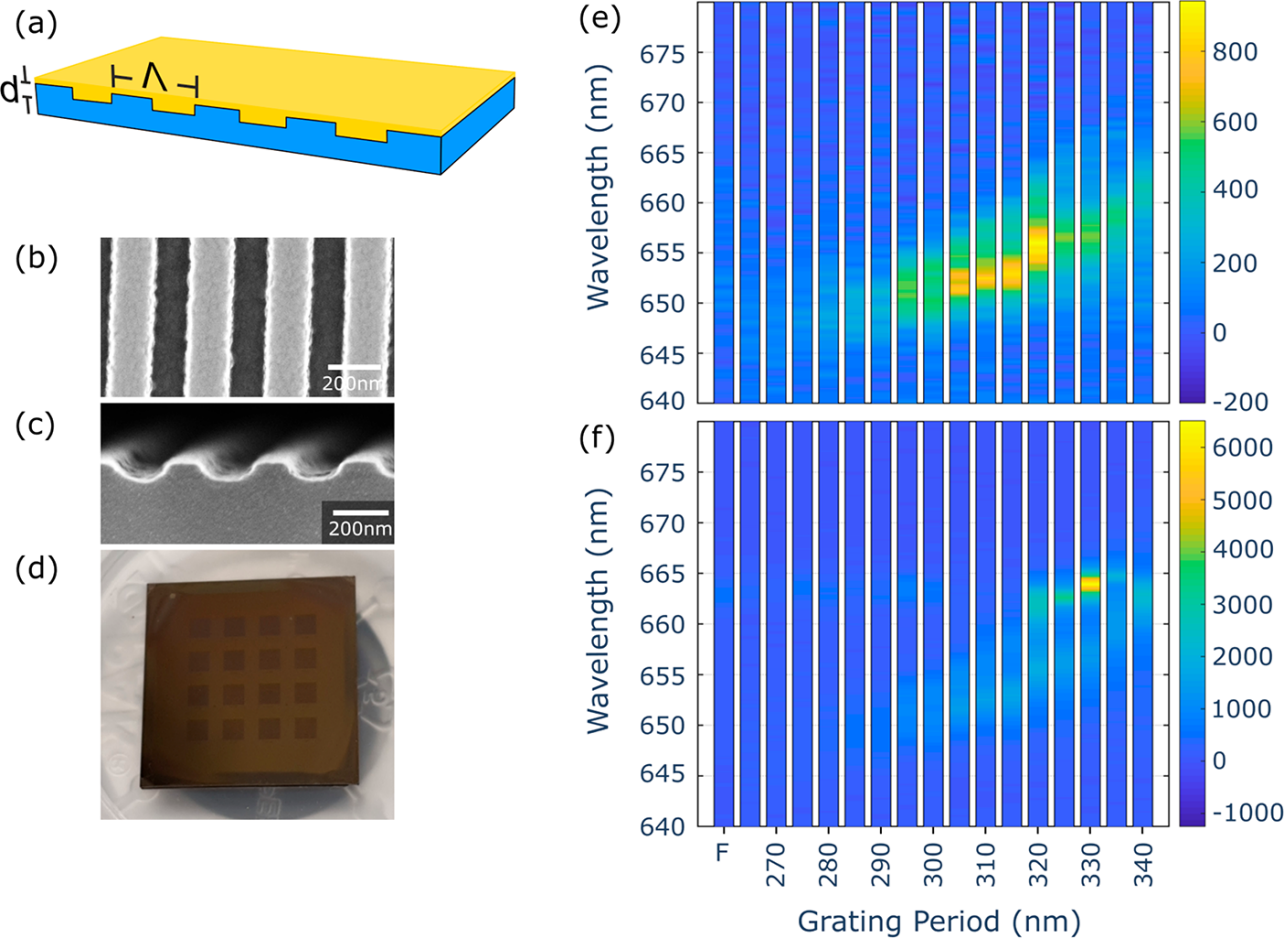
The characterization of the PEA_2_SnI_4_ DFB, at 77 K, is presented as follows: (a) depicts a DFB device, which consists of a spin coated PEA_2_SnI_4_ film (yellow) and a Si/SiO_2_ periodic grating (blue); (b) and (c) are the SEM images of the DFB grating as seen from the top and the side, respectively; (d) is a photo of the measured sample, which consists of a 4 × 4 matrix of PEA_2_SnI_4_ DFBs with periodicities ranging from 340 nm to 265 nm; (e) and (f) are the fluence-dependent measurements above and below threshold, respectively. ‘F’ indicates the perovskite film at an unstructured position of the sample.

To assess the lasing potential of PEA_2_SnI_4_, we fabricated a DFB device, consisting of a periodic grating and an active medium, providing distributed reflections and optical gain, respectively (Figure 4a). To achieve an overlap between the gain spectrum of PEA_2_SnI_4_ (Figure 3d) and the resonance wavelength of the DFB, the grating period can be determined according to the Bragg condition: *λ*_B_ = 2Λ*n*_eff_/*m*, where *λ*_B_ is the resonance wavelength, Λ is the grating period, *n*_eff_ is the effective refractive index and *m* is the grating order.^54^ To obtain a surface-emitting DFB device, we worked with a second-order grating, corresponding to *m* = 2. For the DFB design, we considered a perovskite refractive index of 2.5 at 666 nm, which is the value extracted from ellipsometry measurements (Figure S13). The effective refractive index *n*_eff_ was estimated using a slab waveguide approximation, consisting of three layers: air, the perovskite film, and SiO_2_, where the perovskite was embedded between the other two layers. To have a more precise estimation of the DFB performance, we implemented a finite element method (FEM) based model. This provided information about the change of the DFB resonance wavelength as a function of the grating period (Λ), grating depth (*h*_g_), and the thickness of the perovskite film (*h*_wg_) (Figure S14). The simulations indicate that the resonance wavelength is highly sensitive to parameter changes, which becomes even more relevant given the narrow bandwidth of the PEA_2_SnI_4_ gain spectrum. To account for this, we fabricated and measured a variety of grating periodicities. The DFBs were fabricated from silicon substrates with a 1.5 μm SiO_2_ cladding layer and patterned using electron beam lithography and reactive ion etching (Figure 4b,c). Afterward, the perovskite film, with a thickness of about 110 nm, was spin coated on top of the DFB grating (Figure 4d). A total of 16 different DFBs were patterned on a single sample, each having a different grating periodicity ranging from 265 nm to 340 nm, in steps of 5 nm. Fluence-dependent measurements were carried out at 77 K for the 16 DFB devices as well as for the bare PEA_2_SnI_4_ film (Figure 4e,f). Below threshold (Figure 4e), the DFB gratings give rise to an enhanced spontaneous emission around 651–657 nm, which corresponds to the excitonic peak of PEA_2_SnI_4_ centered around 654 nm. The PL enhancement shifts in accordance with the change in the periodicity of the DFBs, indicating a good optical coupling with the resonator. Otherwise, above threshold (Figure 4f), the highest intensity is obtained from the grating with a periodicity of 330 nm, where the enhanced signal dominates over the bare film and the other grating periodicities, which indicates optimal spectral matching for the 330 nm grating. Similarly to what can be observed below threshold, the above threshold spectra show a shift of the signal enhancement as determined by the period of the DFB gratings.

**Figure 5:**
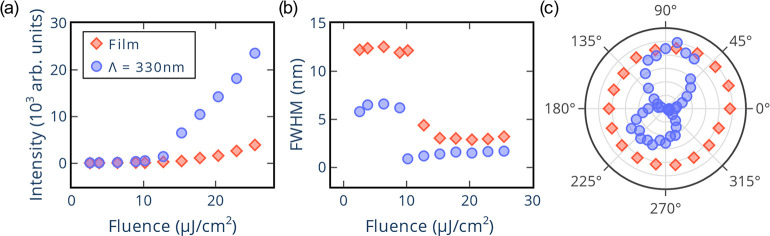
A comparison, at 77 K, between the bare PEA_2_SnI_4_ film and the best performing PEA-DFB device (periodicity of 330 nm): (a) is the output intensity as a function of excitation fluence; (b) shows the fwhm as a function of excitation fluence; and (c) is the change of output intensity as a function of the polarization angle.

The features, at 77 K, of the bare PEA_2_SnI_4_ film (PEA-film) and the film deposited on the DFB with a period of 330 nm (PEA-DFB device) are compared in Figure 5, showing the difference between a system with and without a resonator. This is important given that the presence of a resonator, which provides feedback, plays a key role in defining the properties of the emitted light. For example, in a laser, feedback gives rise to amplified light that is highly directional, coherent, and narrow in bandwidth. In contrast, to obtain ASE, feedback is not required, consequently, ASE possesses features that resemble those of lasing, however less sharp, such as a lower directionality, a broader bandwidth, and a softer threshold behavior. As can be observed in Figure 5a, there is a reduction of the intensity threshold for the PEA-DFB device compared to the PEA-film from 29 μJ/cm^2^ to 19 μJ/cm^2^. Moreover, before saturation, the PEA-DFB device shows an intensity enhancement of about 1 order of magnitude in comparison to the PEA film. In addition, the fwhm of the PEA film narrows by a factor of 4, from 12 nm to 3 nm, while the PEA-DFB device has a more significant decrease by a factor of about seven, from 6.6 nm to 0.9 nm (Figure 5b), as well as a faster line width reduction than the one seen with the PEA film. Moreover, unlike the PEA film, the PEA-DFB device presents an evident polarization dependence (Figure 5c), which is a feature imprinted by the cavity mode of the DFB resonator. In summary, the data from Figure 5 reveal the synergistic effects, induced by the geometrical and physical properties of the PEA-DFB device, suggesting lasing action.^55,56^ Furthermore, even under room temperature conditions, the excitation fluence needed to reach the onset of amplification with the PEA-DFB device is about half of the one required with the bare film (Figure S15). Overall, the results obtained with our device indicate that 2D tin perovskites are promising gain media.

## 3 Conclusions

In summary, our work sheds light on the ASE characteristics of layered perovskites, which have remained elusive for the last two decades. We found that the optical gain properties of 2D perovskites can be tuned and improved by modifying their chemical composition. The flexible alkyl BA cations support the growth of highly defective systems unable to sustain ASE. Meanwhile, the aromatic cations PEA and NMA promote the formation of more rigid perovskite lattices having improved film morphologies with large and compact crystalline grains. On the one hand, the intrinsic photoinstability of NMA_2_SnI_4_ hindered its ASE operation. On the other hand, PEA_2_SnI_4_ was found to be a notable gain medium given its low defectivity, high optical quality, and stability. In fact, it was possible to successfully integrate PEA_2_SnI_4_ in an optically pumped DFB lasers, owing to its low-threshold ASE and optical gain beyond 4000 cm^–1^ at 77 K. This work highlights the potential of 2D tin perovskites for lasing applications and provides fundamental knowledge for making the best use of low-dimensional layered perovskites as optical gain media. Our work also underlines the importance of defect passivation strategies, which should be taken into account to further improve the performance of perovskites. Finally, chemical design of the spacer cations aimed at increasing the structural rigidity of the perovskite will play a critical role in minimizing ASE thermal quenching and in achieving room temperature ASE operational with these excitonic systems.

## 4 Experimental Section/Methods

### 4.1 Synthesis of 1-Naphthylmethylammonium Iodide (NMA)I

1-Naphthylmethylamine (1.5 mL, 0.01 mmol) was dissolved in 40 mL of tetrahydrofuran (THF) and 3 equiv of HI (57% water solution, stabilized) were added dropwise to the solution kept in an ice bath under magnetic stirring. After 3 h the reaction was stopped, and the product precipitated from THF by adding dichloromethane (DCM). The washing procedure was repeated 4 times, and the final product (NMA)I was collected as a white powder by drying it under vacuum at 60 °C in a rotary evaporator.

### 4.2 Perovskite Synthesis

For the synthesis of BA_2_SnI_4_, PEA_2_SnI_4_, and NMA_2_SnI_4_, the organic precursors BAI, PEAI, and NMAI were mixed with SnI_2_ in 2:1 molar ratio in dimethylformamide (DMF), giving a concentration of 0.2 M. The mixture was heated at 100 °C for 1 h and then filtered (PTFE filters, 0.45 μm). Substrates (glass or patterned Si/SiO_2_ wafers) were cleaned by sonication in acetone, deionized water, and isopropanol followed by an oxygen plasma treatment. The hot solution (100 °C) was dropped on the glass substrate and spin coated at 5000 rpm for 30 s. The films were annealed at 100 °C for 15 min.

To prevent sample degradation under ambient air, once the perovskite films were fabricated in the glovebox, each sample was stored in a nitrogen-filled container and subsequently placed inside a plastic vacuum sealed bag. Furthermore, the measurements were carried out in vacuum.

### 4.3 ASE and VSL Measurements

The samples were excited with a pulsed 532 nm green laser (Innolas Picolo second harmonic), having a pulse duration of 800 ps and a repetition rate of 1 kHz. For the ASE measurements, the laser signal was focused on the sample with a 10 cm spherical lens. Moreover, to describe the behavior of the photoluminescence spectra as a function of excitation fluence, the intensities and line width were extracted as follows: (1) below threshold, the emission closest to the ASE peak was fitted to a Gaussian function, and (2) above threshold, the ASE peak was fitted to a Lorentzian curve.

For the VSL measurements, the same laser excitation conditions as for the ASE measurements were used. In addition, a cylindrical lens (*f* = 100 mm) focused the laser beam on a 1 mm slit, resulting in a stripe shaped beam that was imaged on the sample with a biconvex lens (*f* = 50 mm). Furthermore, the length of the excitation stripe was adjusted using a movable slit and the output emission was measured by means of a PL collection line perpendicularly aligned to the excitation plane. To avoid artifacts due to the pump beam spatial shape, the laser beam was enlarged to achieve a 1 mm stripe with a flat-top profile.^26^

For the ASE and VSL measurements a fiber coupled Maya-1000PRO spectrometer was used for detection.

### 4.4 DFB Fabrication

A positive resist (SML 300, EM resist LTD, Macclesfield, UK) was spun (2000 rpm) on a silicon chip and prebaked for 10 min at 180 °C. Afterward, a conductive polymer was spun (4000 rpm) on the sample and prebaked for 2 min at 120 °C. The sample was subsequently exposed with an EBL system (voltage, 50 kV; dose, 750 μC cm^–2^). The exposed sample was developed under ultrasonication in a solution of isopropanol/H_2_O:7/3 for 45 s and descummed for 20 s in an O_2_ plasma. The structuring was performed with an Inductively Coupled Plasma-Reactive Ion Etching (ICP-RIE) tool, where the following parameters were used: reactive gas = CHF_3_ (20 sccm), ICP = 450 W, bias = 182 V, pressure = 0.012 mbar, time = 90 s.

## Supporting Information Available

Experimental details (materials, synthesis, structural and morphological characterization, spectroscopic characterization, photothermal deflection spectroscopy, ASE and VSL measurements, ellipsometry, DFB fabrication) and additional data (XRD, temperature-dependent absorption and photoluminescence, ASE stability, PL stability, temperature-dependent ASE, fluence-dependent ASE, ASE slope and gain as a function of temperature, refractive index, extinction coefficient, DFB-device simulations, PEA-DFB device spectra at room temperature) (PDF)

## References

[ref1] Halide Perovskites for Photonics; Vinattieri A., Giorgi G., Eds.; AIP Publishing: Melville, NY, 2021.

[ref2] Fakharuddin A.; Gangishetty M. K.; Abdi-Jalebi M.; Chin S.-H.; bin Mohd Yusoff A. R.; Congreve D. N.; Tress V.; Deschler F.; Vasilopoulou M.; Bolink H. J. Perovskite Light-Emitting Diodes. Nat. Electron. 2022, 5, 203–216. 10.1038/s41928-022-00745-7.

[ref3] Deschler F.; Price M.; Pathak S.; Klintberg L. E.; Jarausch D.-D.; Higler R.; Hüttner S.; Leijtens T.; Stranks S. D.; Snaith H. J.; Atatüre M.; Phillips R. T.; Friend R. H. High Photoluminescence Efficiency and Optically Pumped Lasing in Solution-Processed Mixed Halide Perovskite Semiconductors. J. Phys. Chem. Lett. 2014, 5, 1421–1426. 10.1021/jz5005285. 26269988

[ref4] Xing G.; Mathews N.; Lim S. S.; Yantara N.; Liu X.; Sabba D.; Grätzel M.; Mhaisalkar S.; Sum T. C. Low-Temperature Solution-Processed Wavelength-Tunable Perovskites for Lasing. Nat. Mater. 2014, 13, 476–480. 10.1038/nmat3911. 24633346

[ref5] Qin C.; Sandanayaka A. S. D.; Zhao C.; Matsushima T.; Zhang D.; Fujihara T.; Adachi C. Stable Room-Temperature Continuous-Wave Lasing in Quasi-2D Perovskite Films. Nature 2020, 585, 53–57. 10.1038/s41586-020-2621-1. 32879501

[ref6] Pourdavoud N.; Haeger T.; Mayer A.; Cegielski P. J.; Giesecke A. L.; Heiderhoff R.; Olthof S.; Zaefferer S.; Shutsko I.; Henkel A.; Becker-Koch D.; Stein M.; Cehovski M.; Charfi O.; Johannes H.-H.; Rogalla D.; Lemme M. C.; Koch M.; Vaynzof Y.; Meerholz K.; Kowalsky W.; Scheer H.-C.; Görrn P.; Riedl T. Room-Temperature Stimulated Emission and Lasing in Recrystallized Cesium Lead Bromide Perovskite Thin Films. Adv. Mater. 2019, 31, 1903717 10.1002/adma.201903717. 31402527

[ref7] Dong H.; Zhang C.; Liu X.; Yao J.; Zhao Y. S. Materials Chemistry and Engineering in Metal Halide Perovskite Lasers. Chem. Soc. Rev. 2020, 49, 951–982. 10.1039/C9CS00598F. 31960011

[ref8] Dong H.; Saggau C. N.; Zhu M.; Liang J.; Duan S.; Wang X.; Tang H.; Yin Y.; Wang X.; Wang J.; Zhang C.; Zhao Y. S.; Ma L.; Schmidt O. G. Perovskite Origami for Programmable Microtube Lasing. Adv. Funct. Mater. 2021, 31, 2109080 10.1002/adfm.202109080.

[ref9] Zhu H.; Fu Y.; Meng F.; Wu X.; Gong Z.; Ding Q.; Gustafsson M. V.; Trinh M. T.; Jin S.; Zhu X.-Y. Lead Halide Perovskite Nanowire Lasers with Low Lasing Thresholds and High Quality Factors. Nat. Mater. 2015, 14, 636–642. 10.1038/nmat4271. 25849532

[ref10] Hong X.; Ishihara T.; Nurmikko A. V. Photoconductivity and Electroluminescence in Lead Iodide Based Natural Quantum Well Structures. Solid State Commun. 1992, 84, 657–661. 10.1016/0038-1098(92)90210-Z.

[ref11] Liang D.; Peng Y.; Fu Y.; Shearer M. J.; Zhawng J.; Zhai J.; Zhang Y.; Hamers R. J.; Andrew T. L.; Jin S. Color-Pure Violet-Light-Emitting Diodes Based on Layered Lead Halide Perovskite Nanoplates. ACS Nano 2016, 10, 6897–6904. 10.1021/acsnano.6b02683. 27336850

[ref12] Sutherland B. R.; Sargent E. H. Perovskite Photonic Sources. Nat. Photonics 2016, 10, 295–302. 10.1038/nphoton.2016.62.

[ref13] Xing G.; Wu B.; Wu X.; Li M.; Du B.; Wei Q.; Guo J.; Yeow E. K. L.; Sum T. C.; Huang W. Transcending the Slow Bimolecular Recombination in Lead-Halide Perovskites for Electroluminescence. Nat. Commun. 2017, 8, 14558 10.1038/ncomms14558. 28239146 PMC5333353

[ref14] Gong X.; Voznyy O.; Jain A.; Liu W.; Sabatini R.; Piontkowski Z.; Walters G.; Bappi G.; Nokhrin S.; Bushuyev O.; Yuan M.; Comin R.; McCamant D.; Kelley S. O.; Sargent E. H. Electron-Phonon Interaction in Efficient Perovskite Blue Emitters. Nat. Mater. 2018, 17, 550–556. 10.1038/s41563-018-0081-x. 29760510

[ref15] Fu Y.; Zhu H.; Chen J.; Hautzinger M. P.; Zhu X.-Y.; Jin S. Metal Halide Perovskite Nanostructures for Optoelectronic Applications and the Study of Physical Properties. Nat. Rev. Mater. 2019, 4, 169–188. 10.1038/s41578-019-0080-9.

[ref16] Shi E.; Yuan B.; Shiring S. B.; Gao Y.; Akriti; Guo Y.; Su C.; Lai M.; Yang P.; Kong J.; Savoie B. M.; Yu Y.; Dou L. Two-Dimensional Halide Perovskite Lateral Epitaxial Heterostructures. Nature 2020, 580, 614–620. 10.1038/s41586-020-2219-7. 32350477

[ref17] Huang Y.; Li Y.; Lim E. L.; Kong T.; Zhang Y.; Song J.; Hagfeldt A.; Bi D. Stable Layered 2D Perovskite Solar Cells with an Efficiency of over 19% via Multifunctional Interfacial Engineering. J. Am. Chem. Soc. 2021, 143, 3911–3917. 10.1021/jacs.0c13087. 33660986

[ref18] Cortecchia D.; Dewi H. A.; Yin J.; Bruno A.; Chen S.; Baikie T.; Boix P. P.; Grätzel M.; Mhaisalkar S.; Soci C.; Mathews N. Lead-Free MA_2_CuCl_x_Br_4-x_ Hybrid Perovskites. Inorg. Chem. 2016, 55, 1044–1052. 10.1021/acs.inorgchem.5b01896. 26756860

[ref19] Kondo T.; Azuma T.; Yuasa T.; Ito R. Biexciton Lasing in the Layered Perovskite-Type Material (C_6_H_13_NH_3_)_2_PbI_4_. Solid State Commun. 1998, 105, 253–255. 10.1016/S0038-1098(97)10085-0.

[ref20] Chong W. K.; Thirumal K.; Giovanni D.; Goh T. W.; Liu X.; Mathews N.; Mhaisalkar S.; Sum T. C. Dominant Factors Limiting the Optical Gain in Layered Two-Dimensional Halide Perovskite Thin Films. Phys. Chem. Chem. Phys. 2016, 18, 14701–14708. 10.1039/C6CP01955B. 27184073

[ref21] Leyden M. R.; Matsushima T.; Qin C.; Ruan S.; Ye H.; Adachi C. Amplified Spontaneous Emission in Phenylethylammonium Methylammonium Lead Iodide Quasi-2D Perovskites. Phys. Chem. Chem. Phys. 2018, 20, 15030–15036. 10.1039/C8CP02133C. 29789829

[ref22] Liang Y.; Shang Q.; Wei Q.; Zhao L.; Liu Z.; Shi J.; Zhong Y.; Chen J.; Gao Y.; Li M.; Liu X.; Xing G.; Zhang Q. Lasing from Mechanically Exfoliated 2D Homologous Ruddlesden-Popper Perovskite Engineered by Inorganic Layer Thickness. Adv. Mater. 2019, 31, 1903030 10.1002/adma.201903030. 31408551

[ref23] Booker E. P.; Price M. B.; Budden P. J.; Abolins H.; del Valle-Inclan Redondo Y.; Eyre L.; Nasrallah I.; Phillips R. T.; Friend R. H.; Deschler F.; Greenham N. C. Vertical Cavity Biexciton Lasing in 2D Dodecylammonium Lead Iodide Perovskites. Adv. Opt. Mater. 2018, 6, 1800616 10.1002/adom.201800616.

[ref24] Zhang H.; Hu Y.; Wen W.; Du B.; Wu L.; Chen Y.; Feng S.; Zou C.; Shang J.; Jin Fan H.; Yu T. Room-Temperature Continuous-Wave Vertical-Cavity Surface-Emitting Lasers Inorganic Hybrid Perovskites. APL Mater. 2021, 9, 071106 10.1063/5.0052458.

[ref25] Raghavan C. M.; Chen T.-P.; Li S.-S.; Chen W.-L.; Lo C.-Y.; Liao Y.-M.; Haider G.; Lin C.-C.; Chen C.-C.; Sankar R.; Chang Y.-M.; Chou F.-C.; Chen C.-W. Low-Threshold Lasing from 2D Homologous Organic-Inorganic Hybrid Ruddlesden-Popper Perovskite Single Crystals. Nano Lett. 2018, 18, 3221–3228. 10.1021/acs.nanolett.8b00990. 29694049

[ref26] Alvarado-Leaños A. L.; Cortecchia D.; Folpini G.; Kandada A. R. S.; Petrozza A. Optical Gain of Lead Halide Perovskites Measured via the Variable Stripe Length Method: What We Can Learn and How to Avoid Pitfalls. Adv. Opt. Mater. 2021, 9, 2001773 10.1002/adom.202001773.

[ref27] Kagan C. R.; Mitzi D. B.; Dimitrakopoulos C. D. Organic-Inorganic Hybrid Materials as Semiconducting Channels in Thin-Film Field-Effect Transistors. Science 1999, 286, 945–947. 10.1126/science.286.5441.945. 10542146

[ref28] Matsushima T.; Mathevet F.; Heinrich B.; Terakawa S.; Fujihara T.; Qin C.; Sandanayaka A. S. D.; Ribierre J.-C.; Adachi C. N-Channel Field-Effect Transistors with an Organic-Inorganic Layered Perovskite Semiconductor. Appl. Phys. Lett. 2016, 109, 253301 10.1063/1.4972404.

[ref29] Zhu H.; Liu A.; Shim K. I.; Hong J.; Han J. W.; Noh Y.-Y. High-Performance and Reliable Lead-Free Layered-Perovskite Transistors. Adv. Mater. 2020, 32, 2002717 10.1002/adma.202002717. 32584475

[ref30] Yuan F.; Zheng X.; Johnston A.; Wang Y.-K.; Zhou C.; Dong Y.; Chen B.; Chen H.; Fan J. Z.; Sharma G.; Li P.; Gao Y.; Voznyy O.; Kung H.-T.; Lu Z.-H.; Bakr O. M.; Sargent E. H. Color-Pure Red Light-Emitting Diodes Based on Two-Dimensional Lead-Free Perovskites. Sci. Adv. 2020, 6, eabb0253 10.1126/sciadv.abb0253. 33055155 PMC7556835

[ref31] Lanzetta L.; Marin-Beloqui J. M.; Sanchez-Molina I.; Ding D.; Haque S. A. Two-Dimensional Organic Tin Halide Perovskites with Tunable Visible Emission and Their Use in Light-Emitting Devices. ACS Energy Lett. 2017, 2, 1662–1668. 10.1021/acsenergylett.7b00414.

[ref32] Folpini G.; Palummo M.; Cortecchia D.; Moretti L.; Cerullo G.; Petrozza A.; Giorgi G.; Kandada A. R. S. The Effect of Tin Substitution on the Excitonic Properties of Two Dimensional Metal Halide Perovskites. ChemRxiv 2021, 10.26434/chemrxiv.14330018.v1 (accessed November 9, 2022).

[ref33] Gwinner M. C.; Khodabakhsh S.; Song M. H.; Schweizer H.; Giessen H.; Sirringhaus H. Integration of a Rib Waveguide Distributed Feedback Structure into a Light-Emitting Polymer Field-Effect Transistor. Adv. Funct. Mater. 2009, 19, 1360–1370. 10.1002/adfm.200801897.

[ref34] Wallikewitz B. H.; de la Rosa M.; Kremer J. H.-W. M.; Hertel D.; Meerholz K. A Lasing Organic Light-Emitting Diode. Adv. Mater. 2010, 22, 531–534. 10.1002/adma.200902451. 20217748

[ref35] Halvarsson M.; Langer V.; Vuorinen S. Determination of the Thermal Expansion of κ-Al_2_O_3_ by High Temperature XRD. Surf. Coat. 1995, 76–77, 358–362. 10.1016/0257-8972(95)02558-8.

[ref36] Kirschner M. S.; Diroll B. T.; Guo P.; Harvey S. M.; Helweh W.; Flanders N. C.; Brumberg A.; Watkins N. E.; Leonard A. A.; Evans A. M.; Wasielewski M. R.; Dichtel W. R.; Zhang X.; Chen L. X.; Schaller R. D. Photoinduced, Reversible Phase Transitions in All-Inorganic Perovskite Nanocrystals. Nat. Commun. 2019, 10, 504 10.1038/s41467-019-08362-3. 30700706 PMC6353988

[ref37] Sekiguchi K.; Takizawa K.; Ando S. Thermal Expansion Behavior of the Ordered Domain in Polyimide Films Investigated by Variable Temperature WAXD Measurements. J. Photopolym. Sci. Technol. 2013, 26, 327–332. 10.2494/photopolymer.26.327.

[ref38] Stoumpos C. C.; Malliakas C. D.; Peters J. A.; Liu Z.; Sebastian M.; Im J.; Chasapis T. C.; Wibowo A. C.; Chung D. Y.; Freeman A. J.; Wessels B. W.; Kanatzidis M. G. Crystal Growth of the Perovskite Semiconductor CsPbBr3: A New Material for High-Energy Radiation Detection. Cryst. Growth Des. 2013, 13, 2722–2727. 10.1021/cg400645t.

[ref39] Takahashi Y.; Obara R.; Nakagawa K.; Nakano M.; Tokita J.-Y.; Inabe T. Tunable Charge Transport in Soluble Organic-Inorganic Hybrid Semiconductors. Chem. Mater. 2007, 19, 6312–6316. 10.1021/cm702405c.

[ref40] Cortecchia D.; Neutzner S.; Yin J.; Salim T.; Kandada A. R. S.; Bruno A.; Lam Y. M.; Martí-Rujas J.; Petrozza A.; Soci C. Structure-Controlled Optical Thermoresponse in Ruddlesden-Popper Layered Perovskites. APL Mater. 2018, 6, 114207 10.1063/1.5045782.

[ref41] Knutson J. L.; Martin J. D.; Mitzi D. B. Tuning the Band Gap in Hybrid Tin Iodide Perovskite Semiconductors Using Structural Templating. Inorg. Chem. 2005, 44, 4699–4705. 10.1021/ic050244q. 15962978

[ref42] Singh S.; Li C.; Panzer F.; Narasimhan K. L.; Graeser A.; Gujar T. P.; Kohler A.; Thelakkat M.; Huettner S.; Kabra D. Effect of Thermal and Structural Disorder on the Electronic Structure of Hybrid Perovskite Semiconductor CH_3_NH_3_PbI_3_. J. Phys. Chem. Lett. 2016, 7, 3014–3021. 10.1021/acs.jpclett.6b01207. 27435936

[ref43] Wu X.; Tan L. Z.; Shen X.; Hu T.; Miyata K.; Trinh M. T.; Li R.; Coffee R.; Liu S.; Egger D. A.; Makasyuk I.; Zheng Q.; Fry A.; Robinson J. S.; Smith M. D.; Guzelturk B.; Karunadasa H. I.; Wang X.; Zhu X.; Kronik L.; Rappe A. M.; Lindenberg A. M. Light-induced Picoseconds Rotational Disordering of the Inorganic Sublattice in Hybrid Perovskites. Sci. Adv. 2017, 3, e1602388 10.1126/sciadv.1602388. 28782016 PMC5529057

[ref44] Fang H.-H.; Yang J.; Tao S. X.; Adjokatse S.; Kamminga M. E.; Ye J.; Blake G. R.; Even J.; Loi M. A. Unravelling Light-Induced Degradation of Layered Perovskite Crystals and Design of Efficient Encapsulation for Improved Photostability. Adv. Funct. Mater. 2018, 28, 1800305 10.1002/adfm.201800305.

[ref45] Ueda T.; Shimizu K.; Ohki H.; Okuda T. Z. ^13^C CP/MAS NMR Study of the Layered Compounds [C_6_H_5_CH_2_CH_2_NH_3_]_2_[CH_3_NH_3_]_n_ - _1_Pb_n_l_3n+1_ (n = 1, 2). Z. Naturforsch. 1996, 51, 910–914. 10.1515/zna-1996-0805.

[ref46] Folpini G.; Cortecchia D.; Petrozza A.; Kandada A. R. S. The Role of a Dark Exciton Reservoir in the Luminescence Efficiency of Two-Dimensional Tin Iodide Perovskites. J. Mater. Chem. C 2020, 8, 10889–10896. 10.1039/D0TC01218A.

[ref47] Brenner P.; Bar-On O.; Jakoby M.; Allegro I.; Richards B. S.; Paetzold U. W.; Howard I. A.; Scheuer J.; Lemmer U. Continuous Wave Amplified Spontaneous Emission in Phase-Stable Lead Halide Perovskites. Nat. Commun. 2019, 10, 988 10.1038/s41467-019-08929-0. 30816111 PMC6395683

[ref48] Kazes M.; Oron D.; Shweky I.; Banin U. Temperature Dependence of Optical Gain in CdSe/ZnS Quantum Rods. J. Phys. Chem. C 2007, 111, 7898–7905. 10.1021/jp070075q.

[ref49] Qin L.; Lv L.; Li C.; Zhu L.; Cui Q.; Hu Y.; Lou Z.; Teng F.; Hou Y. Temperature Dependent Amplified Spontaneous Emission of Vacuum Annealed Perovskite Films. RSC Adv. 2017, 7, 15911–15916. 10.1039/C7RA01155E.

[ref50] Delfyett P. J. Laser, Semiconductors. In Encyclopedia of Physical Science and Technology - Lasers and Masers; Meyer R. A., Ed.; Academic Press: Cambridge, MA, USA, 2001; pp 443–475.

[ref51] Sebald K.; Michler P.; Gutowski J.; Kröger R.; Passow T.; Klude M.; Hommel D. Optical Gain of CdSe Quantum Dot Stacks. Phys. Status Solidi A 2002, 190, 593–597. 10.1002/1521-396X(200204)190:2<593::AID-PSSA593>3.0.CO;2-4.

[ref52] Akahane K.; Yamamoto N.; Kawanishi T. High Characteristic Temperature of Highly Stacked Quantum-Dot Laser for 1.55-m Band. IEEE Photon. Technol. Lett. 2010, 22, 103–105. 10.1109/LPT.2009.2035821.

[ref53] Even J.; Wang C.; Grillot F. From Basic Physical Properties of InAs/InP Quantum Dots to State-of-the-Art Lasers for 1.55 μm Optical Communications. In Semiconductor Nanocrystals and Metal Nanoparticles; Chen T., Liu Y., Eds.; CRC Press: Boca Raton, FL, USA, 2016; pp 95–120.

[ref54] Saleh B. E. A.; Teich M. C. Fundamentals of Photonics; Wiley, 2019.

[ref55] Mathies F.; Brenner P.; Hernandez-Sosa G.; Howard I. A.; Paetzold U. W.; Lemmer U. Inkjet-Printed Perovskite Distributed Feedback Lasers. Opt. Express 2018, 26, A144–A152. 10.1364/OE.26.00A144. 29401904

[ref56] Brenner P.; Stulz M.; Kapp D.; Abzieher T.; Paetzold U. W.; Quintilla A.; Howard I. A.; Kalt H.; Lemmer U. Highly Stable Solution Processed Metal-Halide Perovskite Lasers on Nanoimprinted Distributed Feedback Structures. Appl. Phys. Lett. 2016, 109, 141106 10.1063/1.4963893.

