# Supplementary material for: Lasing in Two-Dimensional Tin Perovskites
Source: ACS Nano. 2022 Nov 24;16(12):20671–9. doi: 10.1021/acsnano.2c07705 (PMC9798858; doi:10.1021/acsnano.2c07705)
Supplement: Supplementary file 1 — nn2c07705_si_001.pdf [file nn2c07705_si_001.pdf]

Lasing in two-dimensional tin perovskites

Ada Lilí Alvarado-Leaños,[1,2] Daniele Cortecchia,[1*#] Christian Niclaas Saggau,[3] Samuele Martani,[1] Giulia Folpini,[1] Elena Feltri,[1,2] Munirah D. Albaqami[4], Libo Ma[3] and Annamaria Petrozza[1*]

[1] *Istituto Italiano de Tecnologia, Centre for Nano Science and Technology (CNST@PoliMi), Milan, 20133, Italy*
[2]*Physics Department, Politecnico di Milano, Piazza Leonardo da Vinci 32, 20133 Milano, Italy*
[3] *Institute for Integrative Nanosciences, Leibniz IFW Dresden, Dresden 01069, Germany*
[4]*Chemistry Department, College of Science, King Saud University, Riyadh 11451, Saudi Arabia*

*Annamaria.Petrozza@iit.it; Daniele.Cortecchia2@unibo.it

1 Experimental method

1.1 Materials.

1-Naphtylmethylamine (97%, Sigma Aldrich), tin iodide SnI_2 (TCI), butylammonium iodide BAI (GreatCell Solar), phenethylammonium iodide PEAI (GreatCell Solar), dimethylformamide DMF (anhydrous, Sigma Aldrich), hydriodic acid HI (57% in water, stabilized), tetrahydrofuran THF (99.9%, Sigma Aldrich), dichloromethane DCM (anhydrous, Sigma Aldrich).

1.2 Synthesis of 1-naphtylmethylammonium iodide (NMA)I.

1-Naphtylmethylamine (1.5 ml, 0.01 mmol) was dissolved in 40 ml of tetrahydrofuran (THF) and 3 equivalents of HI (57% water solution, stabilized) were added dropwise to the solution kept in an ice bath under vigorous magnetic stirring. After 3h the reaction was stopped, and the product precipitated from THF by adding dichloromethane (DCM). The washing procedure was repeated 4 times, and the final product (NMA)I was collected in form of a white powder by drying it under vacuum at 60 °C in a rotary evaporator.

1.3 Perovskite synthesis.

For the synthesis of the perovskites BA_2SnI_4, PEA_2SnI_4, NMA_2PbI_4, the organic precursors BAI, PEAI, NMAI were mixed with SnI_2 in 2:1 molar ratio in dimethylformamide (DMF) to achieve the concentration of 0.2M. The mixture was heated at 100 °C for 1h and then filtered with PTFE filters (0.45 μm). Separately, the substrates (either glass or patterned Si/SiO2 wafers) were cleaned by sonication in acetone, deionized water and isopropanol and then

treated under oxygen plasma. The hot solution (100 °C) was dropped on the glass substrate and immediately spin coated at 5000 rpm for 30s. During the process, the lid of the spin coater was left open to allow a faster evaporation of the solvent. The films were annealed at 100 °C for 15 min. Solution preparation and film processing were performed in a nitrogen-filled glovebox.

1.4 Structural and morphological characterization.

Perovskite thin films were characterized with X-ray diffraction (XRD) using a BRUKER D8 ADVANCE with Bragg-Brentano geometry, Cu Kα radiation (λ = 1.54056 Å), step increment of 0.02° and 1 s of acquisition time. For temperature-dependent XRD measurements, the sample was placed in a MTC-LOWTEMP chamber, where the temperature is controlled through an AlCr heater and a cold probe flushed with liquid nitrogen. The sample was cooled at the rate of 10 °C/min, with a thermalization of 5 minutes before starting the data acquisition at each selected temperature. The film thickness was measured with a surface profiler Dektak 150 (Veeco) while the film morphology was characterized with a scanning electron microscope (SEM) MIRA3, TESCAN (in this case, the films were prepared on top of conductive ITO/glass to avoid charging effects during the image acquisition).

1.5 Spectroscopic characterization.

The samples were either encapsulated or kept in vacuum during the measurements to prevent oxidation. Steady state absorption spectra were measured on perovskite thin films deposited on glass with a UV/VIS/NIR spectrophotometer Lambda 1050, Perkin Elmer. Room temperature photoluminescence experiments were performed in a NanoLog (Horiba Jobin Yvon). Temperature-dependent experiments were performed with a Linkam Stage HFS350EV-PB4 using a nitrogen-cooled cold finger.

1.6 Photothermal Deflection Spectroscopy

In Photothermal Deflection Spectroscopy (PDS) the absorption spectrum of a thin film can be obtained by monitoring the change of refractive index of the medium surrounding its surface. The sample is submerged in Tetradecafluorohexane and it is illuminated by a monochromatic light provided by a SuperK Extreme supercontinuum laser, coupled with a SuperK SELECT acousto-optic tunable filter (NKT Photonics). The thermal relaxation of the photogenerated carriers creates a thermal gradient in the portion of liquid around the sample's surface. This, in turn, establishes a refractive index gradient (mirage effect) that deflects a He-Ne laser (JDSU)) aligned parallel and in close proximity to the sample's surface. The steering of the He-Ne laser

is measured by a quadrant detector (PDQ80A, Thorlabs). The absorption coefficient, at each wavelength, is proportional to the amplitude of the deflection signal, granting high sensitivity scatter-free detection. The excitation light is modulated by a chopper (4 Hz) to enable lock-in detection (SR830), and by changing its wavelength we retrieve the full absorption spectrum (after normalizing for the power spectrum of the laser). Long-pass filters are used to prevent leakage straylight.

1.7 ASE & VSL measurements

The samples were excited with a pulsed 532 nm green laser (Innolas Picolo 2nd harmonic), having a pulse duration of 800 ps and a repetition rate of 1 kHz. The excitation fluence was controlled with a filter wheel. The emission was detected with a fiber coupled Maya 1000 spectrometer (1 nm resolution). The pump radiation was filtered by using a 550 nm long-pass filter. For the ASE variable pump intensity measurements, the beam was focused using a 10 cm spherical lens. While in the case of the VSL measurement, a cylindrical lens (f = 100 mm) focused the laser beam on a 1 mm slit, resulting in a stripe-shaped beam of adjustable length, that was then imaged on the sample with a biconvex lens (f = 50 mm). The full details of the VSL procedure were previously reported.[26]

1.8 Ellipsometry

The optical constants, namely the refractive index (n) and the extinction coefficient (k), of PEA_2SnI_4 were measured using an ellipsometer (J.A. Woollam M-2000 Ellipsometer). The sample was spin coated on a glass substrate and the ellipsometry spectra were measured in air and in reflection mode. Ellipsometry data were collected in a wavelength range of 370 – 1696 nm, and at angles of incidence of 50-60º, with steps of 5º. The optical constants were extracted by fitting the ellipsometry data with the J.A. Woollam CompleteEASE software.

1.9 DFB fabrication

A positive resist (SML 300, EM resist LTD, Macclesfield, UK), was spun (2000 rpm) onto a 15x15 mm silicon chip with a 1.5 μm wet oxide layer yielding a 300 nm thick resist film. The resist was prebaked for 10 min at 180°C. Following the prebake of the resist, a conductive polymer was spun (4000 rpm) onto the sample and prebaked for 2 min at 120°C. Afterwards the sample was exposed at 50 kV in an EBL system (Voyager, Raith GmbH, Dortmund, Germany) with a dose of 750 μC cm^{-2}. The sample was developed under mild ultrasonication in a solution of isopropanol/H_2O:7/3 for 45s and descummed for 20s in an O_2 plasma (Pico,

Diener, Elbhausen, Germany). The film was then structured in an Inductively Coupled Plasma-Reactive Ion Etching (ICP-RIE) (Plasma Lab 100, Oxford Instruments plc, Abingdon, UK) where CHF_3 (20 sccm) was the reactive gas. ICP: 450 W, bias: 182 V, pressure: 0.012 mbar, time: 90 s.

The 2D simulations were carried out in a 10x20 µm area, where the perovskite (n=2.5) was sandwiched between a SiO_2 (n=1.45) layer and vacuum (n=1). At the SiO_2/perovskite interface there was a square modulation of equal spacing (duty cycle =0.5). A second order scattering boundary condition formed the edges of the simulation to supress back reflection of light. A dipole source (line current) was placed at the centre of the perovskite layer. The dipole oscillated normal to the simulation plane (TE – polarisation) and its frequency was changed from 410 THz to 490 THz with a 0.2 THz step size. During the simulation the field intensity was integrated over a 2 µm long stripe along the perovskite layer with the dipole at its centre. The resonance frequency of the grating could then be retrieved from the recorded spectrum. The mesh size of the grating and the perovskite layer was restricted between 5 - 50 nm and was determined by the software.

2 Supporting tables and figures.

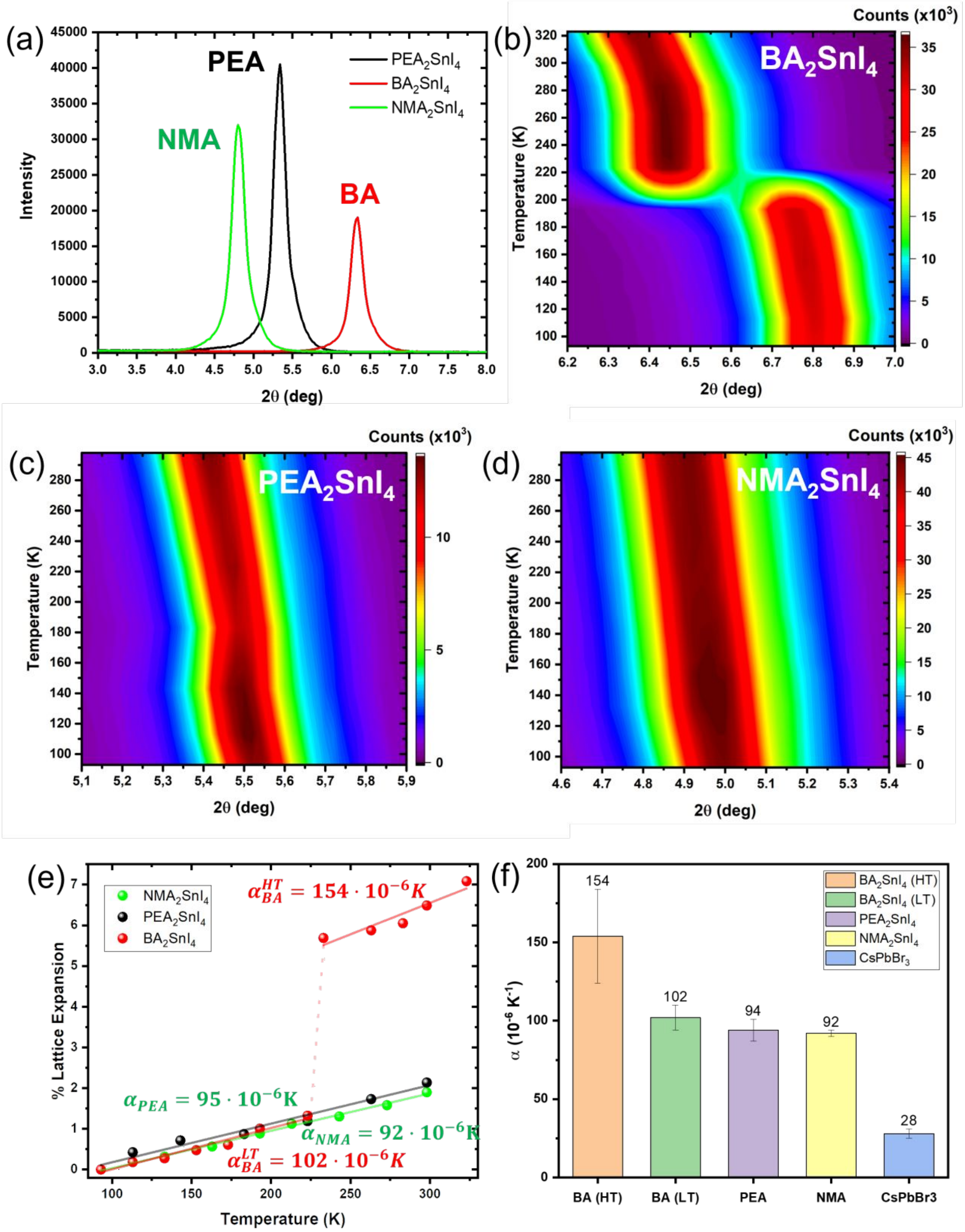

Figure S1. a) Thin film X-Ray diffraction (XRD) of the perovskites. A single diffraction peak for each material is visible due to the strong preferential orientation on the substrate. The peak positions are in good agreement with the expected increase in interplanar spacing increasing the size of the templating cation. Temperature dependent XRD measurements and the observed peak shift for each material due to lattice contraction are shown for b) BA_2SnI_4, c) PEA_2SnI_4 and d) NMA_2SnI_4. e) Expansion of lattice planes (l) versus temperature for the three perovskites. The linear fit of the experimental data yields a thermal expansion coefficient $\alpha = \frac{dl}{l \cdot dT}$ in the

direction orthogonal to the perovskite planes of $\alpha_{BA}^{HT} = 154 \cdot 10^{-6} K^{-1}$ (BA_2SnI_4, high temperature phase), $\alpha_{BA}^{LT} = 102 \cdot 10^{-6} K^{-1}$ (BA_2SnI_4, low temperature phase), $\alpha_{PEA} = 94 \cdot 10^{-6} K^{-1}$ (PEA_2SnI_4), $\alpha_{NMA} = 92 \cdot 10^{-6} K^{-1}$ (NMA_2SnI_4). f) Histogram plot of the linear thermal expansion coefficients in the direction normal to the perovskite planes, as extracted from the XRD thermal dependence analysis. The data for $CsPbBr_3$ are also plotted for comparison (taken from M. S. Kirschner et al, Nat. Commun. 2019, 10, 504).

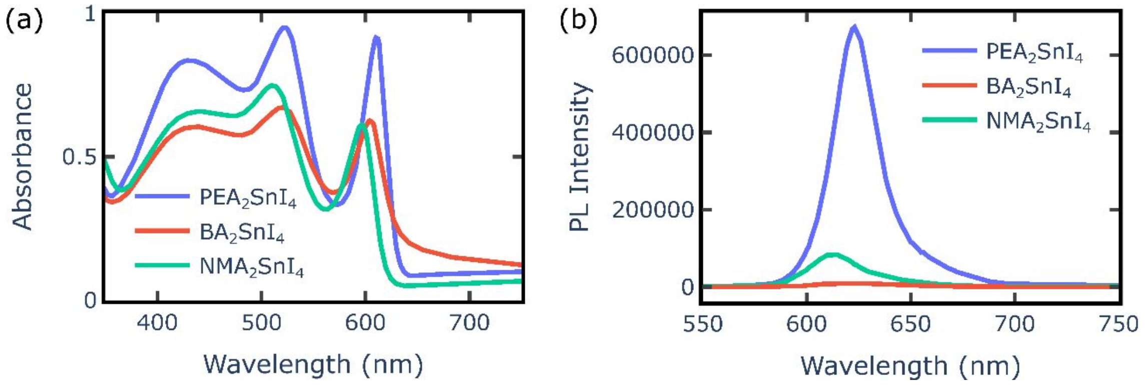

Figure S2. Absorption (a) and photoluminescence spectra (b) of three different perovskites: BA_2SnI_4, PEA_2SnI_4 and NMA_2SnI_4.

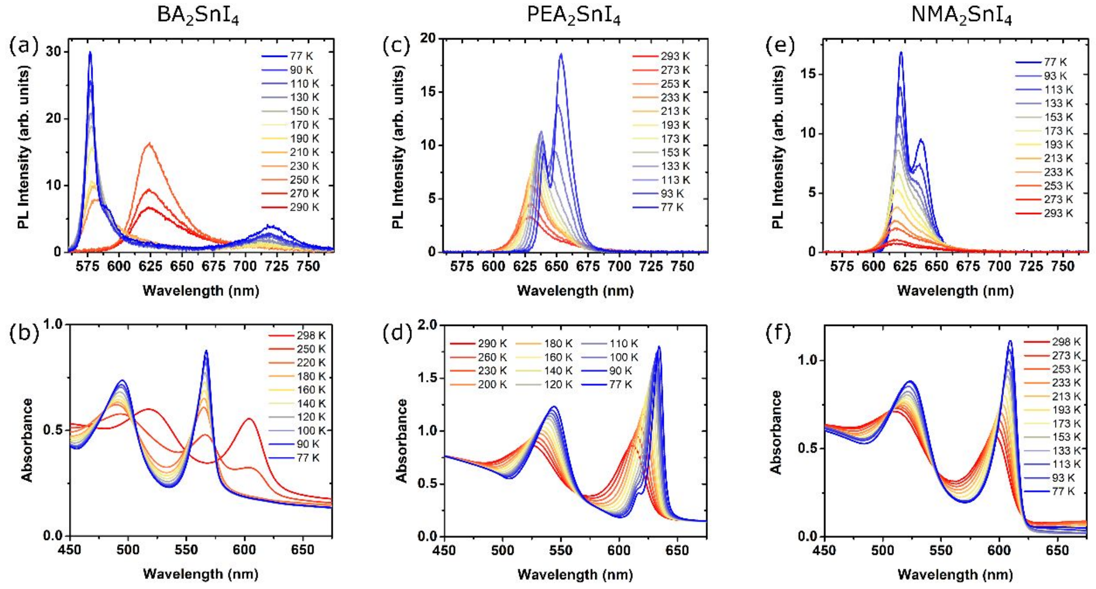

Figure S3. Temperature dependent photoluminescence and absorption of (a,b) BA_2SnI_4, (c,d) PEA_2SnI_4 and (e,f) NMA_2SnI_4.

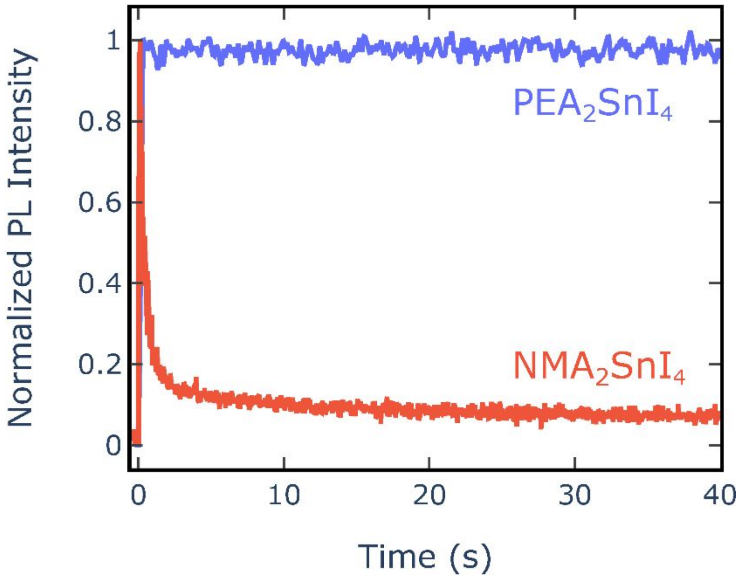

Figure S4. ASE stability of PEA_2SnI_4 and NMA_2SnI_4 under continuous exposure to a 532 nm picosecond laser (pulse duration 800 ps, repetition rate 1KHz) at 284 µJ/cm^2.

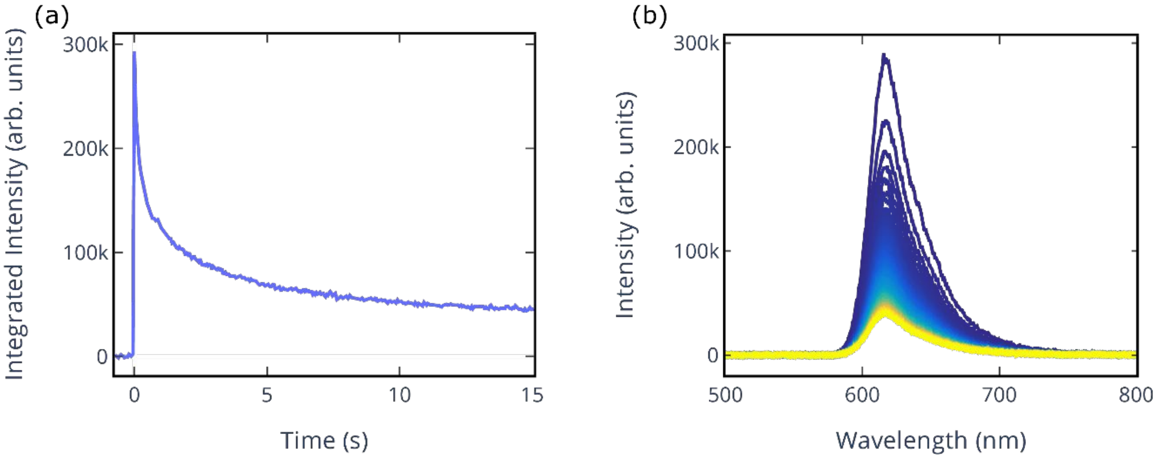

Figure S5. PL stability of NMA_2SnI_4 under continuous exposure to a 532 nm picosecond laser (pulse duration 800 ps, repetition rate 10KHz) at 204 µJ/cm^2.

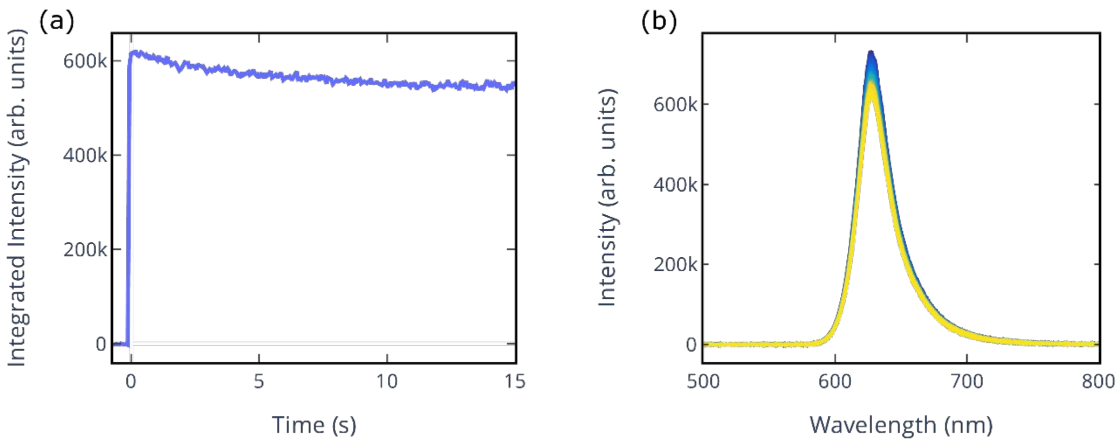

Figure S6. PL stability of PEA_2SnI_4 under continuous exposure to a 532 nm picosecond laser (pulse duration 800 ps, repetition rate 10KHz) at 204 µJ/cm^2.

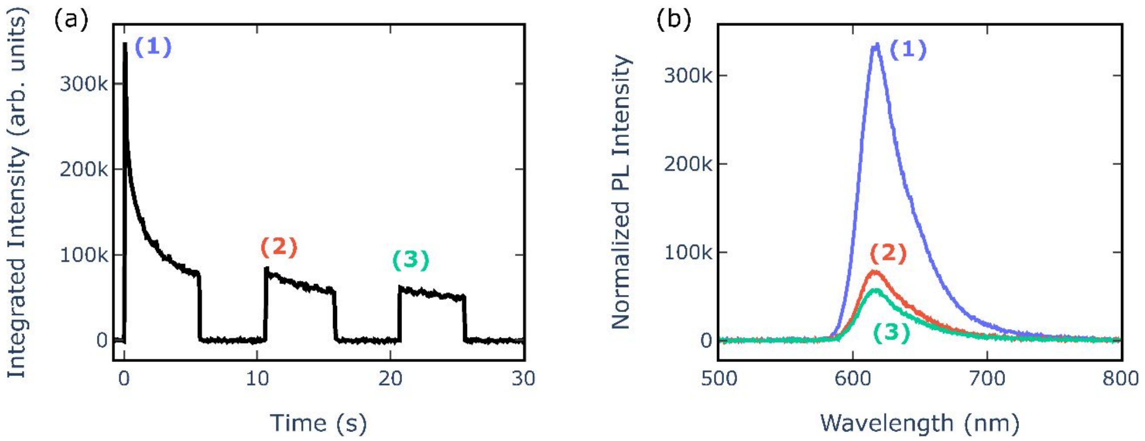

Figure S7. PL stability of NMA_2SnI_4 resulting from a periodic exposure pattern consisting of periods with and without illumination, each lasting about 5 seconds. The excitation was a 532 nm picosecond laser (pulse duration 800 ps, repetition rate 10KHz) at 204 µJ/cm^2.

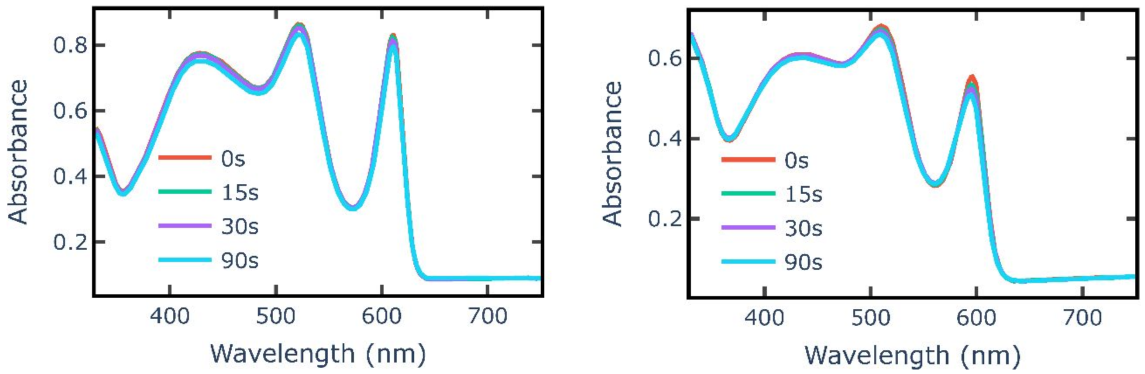

Figure S8. Absorbance of PEA_2SnI_4 for different exposure times (0,15,30 and 90 seconds).

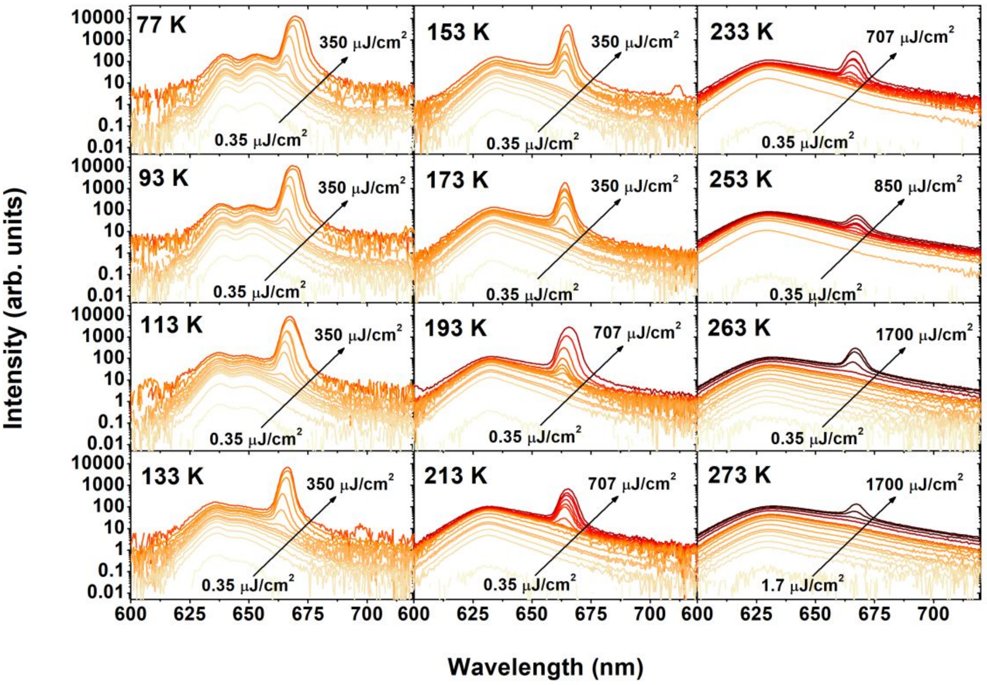

s

Figure S9. ASE evolution at different temperature for PEA_2SnI_4 (excitation wavelength 532 nm, pulse duration 800 ps, repetition rate 1 KHz).

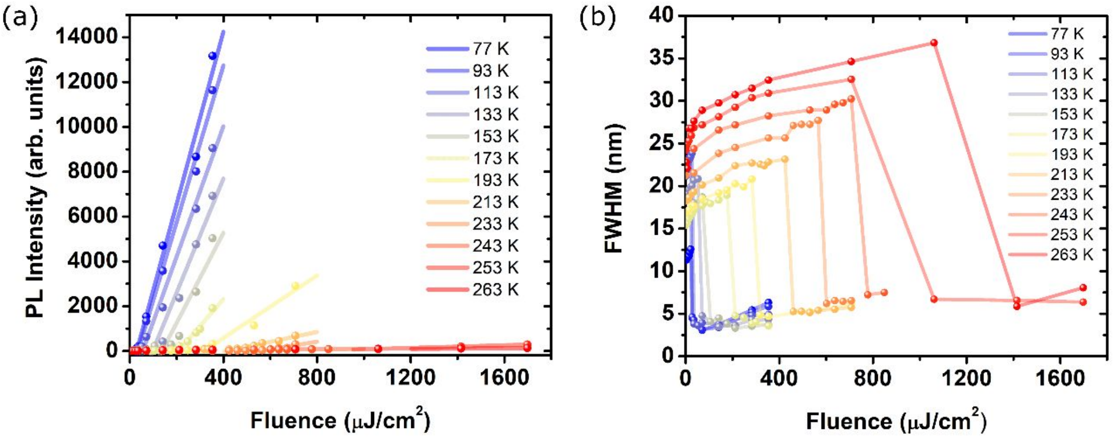

Figure S10. Fluence dependence of the ASE: (a) output intensity and (b) full width at half maximum (FWHM) for PEA_2SnI_4 thin film at different temperatures.

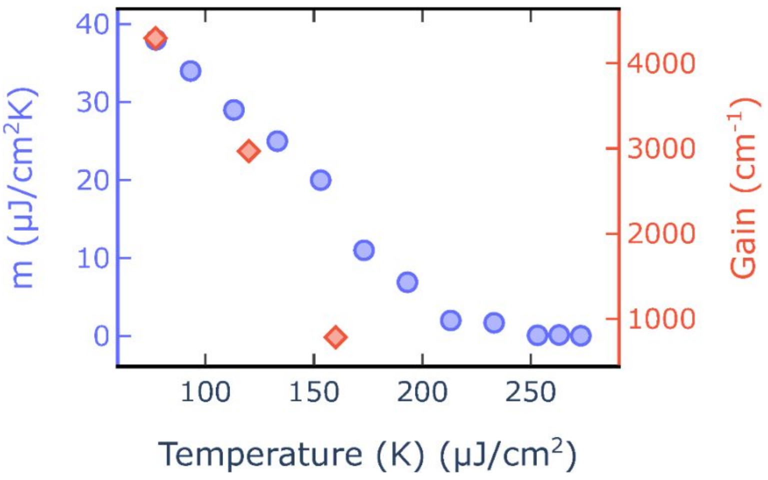

Figure S11. ASE slope (m) and gain as a function of temperature.

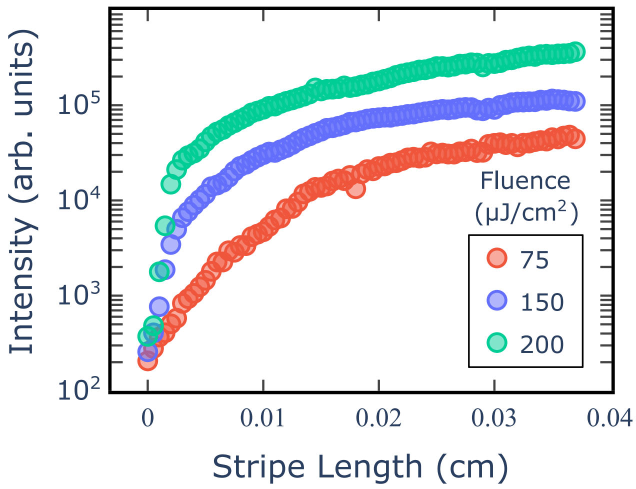

Figure S12. VSL curves for a set of different pump fluences. The curves show the presence of gain as given by the exponential increase of the intensity, followed by a change in slope indicating the saturation of the stimulated emission.

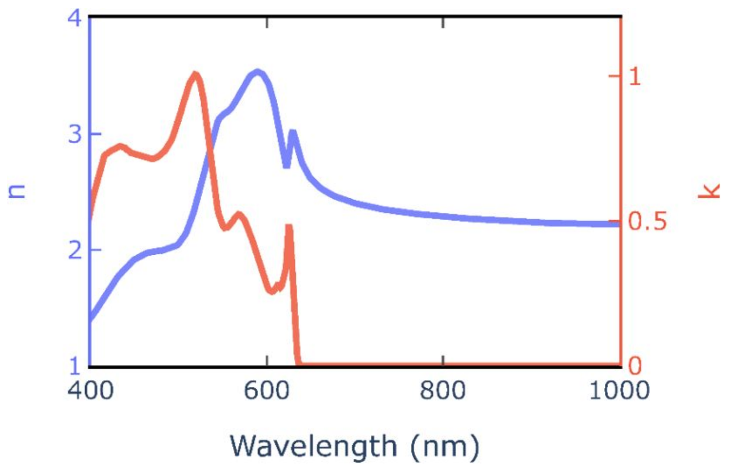

Figure S13. The refractive index (n) and the extinction coefficient (k) of a 100 nm thick PEA_2SnI_4 film, as calculated by ellipsometry.

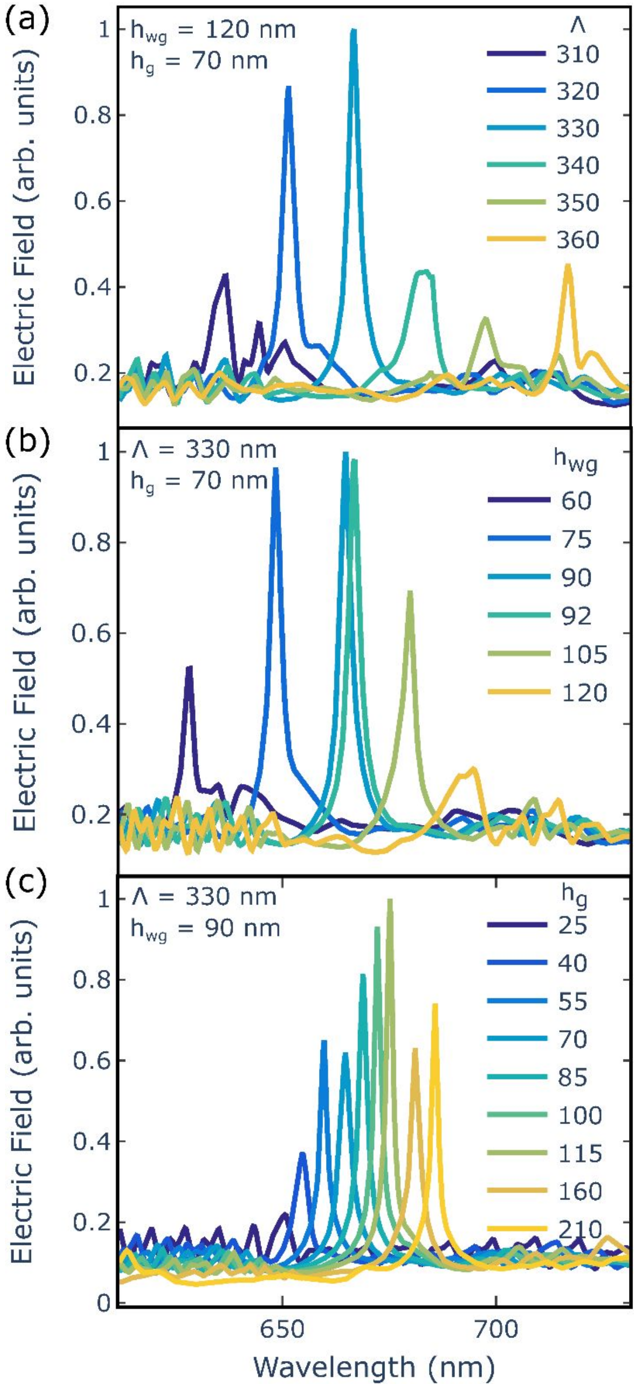

Figure S14. DFB-device simulations, illustrating the influence of the (a) DFB periodicity, (b) perovskite thickness (h_{wg}) and (c) grating depth (h_g).

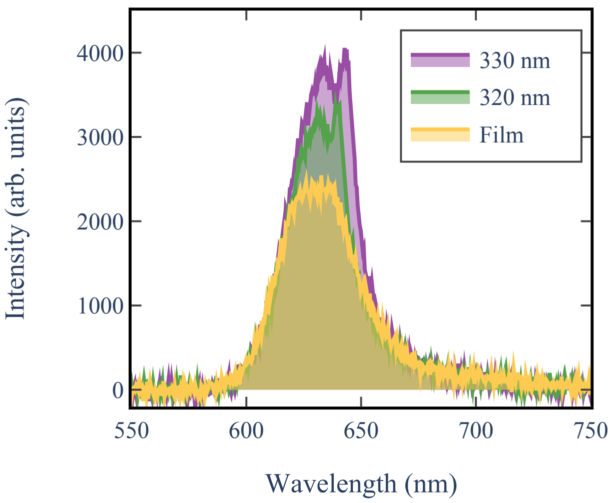

Figure S15. Spectra of the bare PEA_2SnI_4 film (yellow), PEA-DFB device with a periodicity of 320 nm (green), and PEA-DFB device with a periodicity of 330 nm (purple). All spectra were obtained at room temperature with a pump fluence of 1 mJ/cm^2.
